# High CD200 Expression on T CD4+ and T CD8+ Lymphocytes as a Non-Invasive Marker of Idiopathic Pulmonary Hypertension–Preliminary Study

**DOI:** 10.3390/jcm10050950

**Published:** 2021-03-01

**Authors:** Michał Tomaszewski, Ewelina Grywalska, Weronika Topyła-Putowska, Piotr Błaszczak, Marcin Kurzyna, Jacek Roliński, Grzegorz Kopeć

**Affiliations:** 1 Department of Cardiology, Medical University of Lublin, 20-954 Lublin, Poland; tomaszewskimd@gmail.com (M.T.); weronika.topyla@gmail.com (W.T.-P.); 2 Department of Clinical Immunology and Immunotherapy, Medical University of Lublin, 20-093 Lublin, Poland; jacek.rolinski@gmail.com; 3 Department of Cardiology, Cardinal Wyszynski Hospital, 20-718 Lublin, Poland; lubkard@szpital.lublin.pl; 4 Department of Pulmonary Circulation, Thromboembolic Diseases and Cardiology, Centre of Postgraduate Medical Education, European Health Centre Otwock, 05-400 Otwock, Poland; marcin.kurzyna@ecz-otwock.pl; 5 Pulmonary Circulation Center, Department of Cardiac and Vascular Diseases, Faculty of Medicine, Jagiellonian University Medical College, 31-008 Kraków, Poland; grzegorzkrakow1@gmail.com; 6 Department of Cardiac and Vascular Diseases, Centre for Rare Cardiovascular Diseases, John Paul II Hospital, 31-202 Krakow, Poland

**Keywords:** pulmonary arterial hypertension, cardiovascular disease, CD200, CD200R, hypertension immunopathology

## Abstract

Pulmonary arterial hypertension (PAH) can develop subsequently to disorganized endothelial cell proliferation within the pulmonary arteriolar layers that provide mechanical limits to the pulmonary vascular bed. Although the actual factor triggering vascular endothelial proliferation remains unknown to date, genetic susceptibility, hypoxia, inflammation, as well as response to drugs and toxins have been proposed as possible contributors. Since inflammation contributes to vascular remodeling, the changed immune response is increasingly considered a plausible cause of this cardiovascular disease. The interaction of a membrane glycoprotein cluster of differentiation 200 (CD200) and its structurally similar receptor (CD200R) plays a crucial role in the modulation of the inflammatory response. Our previous studies have shown that the overexpression of the other negative co-stimulatory molecule (programmed death cell-PD-1) and its ligand-1 (PD-L1) is closely related to iPAH and the presence of Epstein-Barr virus (EBV) reactivation markers. Therefore, we considered it necessary to analyze the different types of PAH in terms of CD200 and CD200R expression and to correlate CD200/CD200R pathway expression with important clinical and laboratory parameters. The CD200/C200R-signaling pathway has not been subject to much research. We included 70 treatment-naïve, newly diagnosed patients with PAH in our study. They were further divided into subsets according to the pulmonary hypertension classification: chronic thromboembolic pulmonary hypertension (CTEPH) subset, pulmonary arterial hypertension associated with congenital heart disease (CHD-PAH), pulmonary arterial hypertension associated with connective tissue disease (CTD-PAH), and idiopathic pulmonary arterial hypertension (iPAH). The control group consisted of 20 healthy volunteers matched for sex and age. The highest percentages of T CD200+CD4+ and T CD200+CD8+ lymphocytes were observed in the group of patients with iPAH and this finding was associated with the presence of EBV DNA in the peripheral blood. Our assessment of the peripheral blood lymphocytes expression of CD200 and CD200R indicates that these molecules act as negative co-stimulators in the induction and persistence of PAH-associated inflammation, especially that of iPAH. Similar results imply that the dysregulation of the CD200/CD200R axis may be involved in the pathogenesis of several immune diseases. Our work suggests that CD200 and CD200R expression may serve to distinguish between PAH cases. Thus, CD200 and CD200R might be useful as markers in managing PAH and should be further investigated.

## 1. Introduction

Pulmonary arterial hypertension (PAH) is a condition associated with abnormally high blood pressure in the pulmonary artery. PAH has been included by the European Society of Cardiology in the comprehensive clinical classification of pulmonary hypertension (PH) of 2015, alongside pulmonary hypertension due to left heart disease or to lung disease and/or hypoxia, chronic thromboembolic pulmonary hypertension (CTEPH), and other pulmonary artery obstructions, as well as pulmonary hypertension with unclear and/or multifactorial mechanisms [1]. PAH can be classified based on its underlying cause including idiopathic, heritable, drug- and toxin-induced, in addition to its association with other diseases, e.g., connective tissue disease or congenital heart disease.

The diagnosis of PAH is built upon a hemodynamic assessment of pre-capillary pulmonary hypertension. This is defined as an increase in mean pulmonary arterial pressure (mPAP) ≥25 mmHg, normal pulmonary artery wedge pressure (PAWP) ≤ 15 mmHg, and pulmonary vascular resistance (PVR) >3 Wood units (WU) [2]. CTEPH, the clinical manifestation of which mimics that of PAH, is also classified as pre-capillary PH. For that reason, both conditions are usually studied jointly.

PAH can develop as an outcome of disorganized cell proliferation within the pulmonary arteriolar layers that provide mechanical limits to the pulmonary vascular bed. Although the actual factor triggering vascular endothelial proliferation is still unknown, genetic susceptibility, drug and toxin response, hypoxia, and inflammation have been proposed as possible contributors [3]. Since inflammation contributes to vascular remodeling, immune response alteration is increasingly considered to be a plausible cause of this cardiovascular disease [4].

The membrane glycoproteins, differentiation-200 (CD200) and its receptor (CD200R) are grouped within the category of immunoglobulin-like proteins. These mainly exert immunomodulating functions. CD200 is expressed by neurons, vascular endothelial cells, T and B lymphocytes [5], while CD200R is expressed by cells of the myeloid lineage (monocytes, macrophages) and on T and B lymphocytes [6,7]. The interaction between CD200 and CD200R results in the activation of intracellular inhibitors, especially RasGAP, bringing about the inhibition of cell effector functions. Research has revealed that CD200R activation stimulates T lymphocyte differentiation into Treg cells, enhances indoleamine 2,3-dioxygenase (IDO) activation, modulates cytokine release, and stimulates the synthesis of anti-inflammatory cytokines such as IL-10 and TGF-β [8].

Human sample and animal model studies have shown that the reduction of CD200 glycoprotein levels is associated with the inflammatory process and aging. Indeed, supplementation of recombinant, soluble CD200 protein has been noted to reduce the inflammatory response [9,10]. In vitro experimental studies have also demonstrated that suppression of the inflammatory response associated with CD200 is proportional to the level of cellular expression of CD200R [11].

Research has highlighted the role of CD200 in the spread of cancer cells and hypersensitivity reactions [12,13]. These proteins are capable of inducing immune tolerance, as well as regulating cell differentiation, cell adhesion, and chemotaxis [14]. Studies have confirmed that these molecules play a role in the hemostasis of the immune system, and inhibit the inflammatory response to both external antigens (pathogens, allergens, etc.) and internal factors triggering the activation of inflammatory cells (hypoxia, tissue damage, etc.) [15,16,17].

Our previous work has shown that the overexpression of the negative co-stimulatory molecule PD-1 and the PD-L1 ligand is closely related to idiopathic pulmonary arterial hypertension (iPAH) and the presence of Epstein-Barr virus (EBV) reactivation markers [18]. Therefore, we analyzed the different types of PAH in terms of CD200 and CD200R expression and correlated CD200/CD200R pathway expression with important clinical and laboratory parameters. PAH is complex and the CD200/C200R-signaling pathway has not been fully investigated. Hence, the role it plays in PAH development is not well understood. The present study is aimed at investigating CD200 and CD200R expression in the PAH patient blood lymphocytes and comparing this with healthy controls. In addition, we will ascertain their relation to the severity of the disease.

## 2. Materials and Methods

### 2.1. Patients and Controls

We included 70 treatment-naïve, newly diagnosed patients with PAH in our study. They were further divided into subsets according to their pulmonary hypertension classification: CTEPH subset - 10 patients (3 men and 7 women), pulmonary arterial hypertension associated with congenital heart disease (CHD-PAH) - 26 patients (7 men and 19 women), pulmonary arterial hypertension associated with connective tissue disease (CTD-PAH) - 9 patients (9 women), and iPAH - 25 patients (10 men and 15 women). The control group consisted of 20 healthy volunteers (8 men and 12 women) matched for age and sex [18]. European Society of Cardiology criteria were employed for iPAH diagnosis [1]. The enrollees did not receive immunomodulatory treatment, had no signs of infection ≤3 months before enrolment, had not undergone a blood transfusion, or had no autoimmune, neoplastic, or allergic diseases. WHO criteria were employed for determining the functional class of heart failure [1]. Besides a six-minute walk test, each patient underwent complete blood count and natriuretic peptide (BNP) concentration assessments. Basic laboratory tests were accomplished in the ALAB laboratory of the Medical University Clinical Hospital in Lublin. A Phillips iE33 instrument was used for echocardiographic examination of the heart (ECHO). Cardiac catheterization was performed in the Haemodynamics Laboratory of the University Clinical Hospital and the Cardiology Department of the Provincial Specialist Hospital in Lublin. Herein, the hemodynamic assessment standards endorsed by the Polish Cardiac Society were followed [19]. Approval for the study was gained from the Ethics Committee of the Medical University of Lublin (KE-0254/309/2016), and all enrollees gave written informed consent. This study was conducted in accordance with the Helsinki Declaration as described previously [18].

### 2.2. Preparation of Material

We collected 5 mL of peripheral blood in EDTA-coated tubes (Sarstedt, Nümbrecht, Germany) to isolate peripheral blood mononuclear cells (PBMC). Briefly, 5 mL of the whole blood diluted with 5 mL of saline was layered onto 5 mL of Ficoll-Paque™ (Milteny Biotec, Bergisch-Gladbach, Germany) in 15 mm tubes. The tubes were then continuously centrifuged at 400× *g* for 30 min. The PBMC layer was subsequently harvested, and cells were counted and assayed for viability after applying trypan blue (0.4% trypan blue solution; Sigma Aldrich, Hamburg, Germany). Only PBMC with a viability ≥95% were included in further experimentation [17,18].

### 2.3. Immunophenotyping

The peripheral blood (PB) samples were diluted at a 1:1 ratio with 0.9% magnesium (Mg^2+^)/calcium (Ca^2+^) and free phosphate-buffered saline (PBS) (Biochrome AG, Berlin, Germany). The diluted samples were separated through density-gradient centrifugation by layering on 3 mL of Gradisol L (Aqua Medica, Poland; specific gravity 1.077 g/mL) and centrifuging at 700× *g* for 20 min. After PBMC collection using Pasteur pipettes, these were washed twice with Ca^2+^/Mg^2+^-free PBS for 5 min. Subsequently, the cells were withheld in 1 mL of PBS and counted in a Neubauer chamber. Their viability was assessed with the use of Trypan blue (0.4% Trypan Blue Solution, Sigma Aldrich, Temecula, CA, USA).

**Figure 1:**
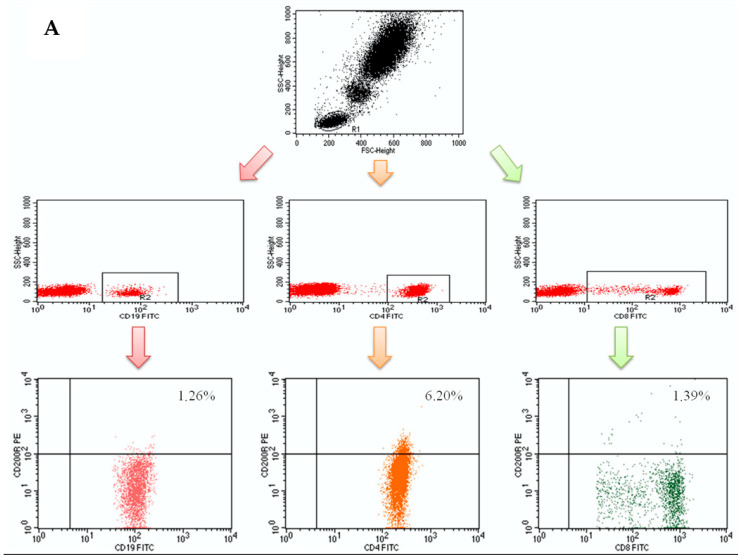

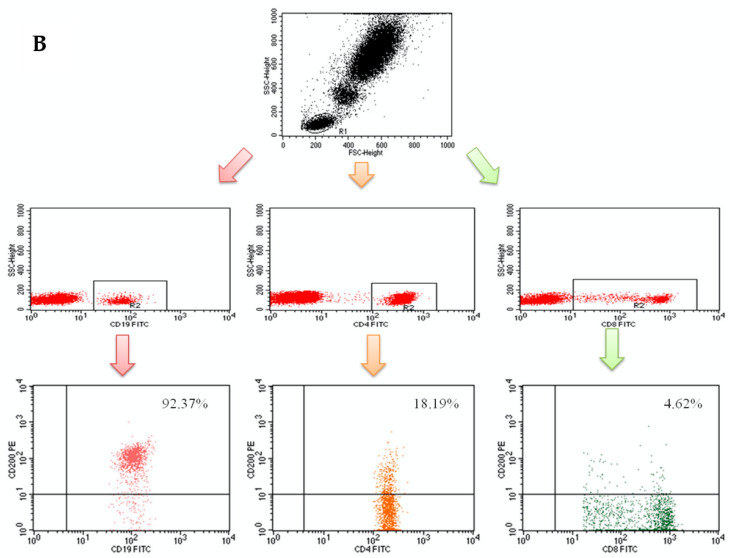
(**A**) Sample analysis of CD200 expression on B and T (CD4+ and CD8+) lymphocytes in idiopathic pulmonary arterial hypertension (iPAH) patients; (**B**) Sample analysis of CD200R expression on B and T (CD4+ and CD8+) lymphocytes in iPAH patients

The percentages of CD200+ and CD200R+ cells among the CD4+ T, CD8+ T, and CD19+ B lymphocyte populations were estimated through flow cytometry. Following PBMC isolation, the cell suspensions were placed into single tubes (1 × 10^6^ cells per sample) and afterward incubated with the pertinent monoclonal antibodies (mAbs). We utilized fluorochrome-conjugated mAbs in opposition to the following markers: mouse anti-human CD19-FITC, mouse anti-human CD3-CyChrome, mouse anti-human CD8-FITC, mouse anti-human CD4-FITC, mouse anti-human CD200-PE, and mouse anti-human CD200R-PE and CD45- fluorescein isothiocyanate (FITC)/CD14- phycoerythrin (PE) (BD Biosciences, San Jose, CA, USA). We also used the Human Treg Flow kit (FOXP3 Alexa Fluor 488/CD4 PE-Cyanine-5 (Cy5)/CD25 PE; BioLegend, San Diego, CA, USA) to recognize the CD4+CD25+high forkhead box P3 (FOXP3+) Treg subpopulation. In the course of the analysis, the CD16+CD56+ NK+ cells and the CD3+CD16+CD56+ natural killer T-like (NKT-like) cell population and were also measured with CD16CD56-PE, anti-CD3-FITC, and CD45- peridinin-chlorophyll-protein (PerCP) mAbs (BD Biosciences, San Jose, CA, USA). The cells were incubated at room temperature for 20 min with 20 μL of each mAb per sample. The suspensions were subsequently washed two times with PBS (700× *g*, 5 min) and thereafter analyzed in the FACSCalibur flow cytometer (Becton-Dickinson, Franklin Lakes, NJ, USA) containing a 488-nm argon laser. Data was collected with the FACS Diva Software 6.1.3 and CellQuest Pro Software (Becton Dickinson, Franklin Lakes, NJ, USA). It included 20,000 cells per run. The cells were labeled and examined according to the lymphocyte gates at combined CD45/CD14 coordinates. The samples were gated on forward scatter vs. side scatter. The flow cytometry analysis results are presented as a percentage of stained cells. The background fluorescence was established with the use of directly conjugated isotype-matched FITC-Immunoglobulin G1 (IgG1) and PE-IgG1 controls to eliminate contamination and cell aggregates [18,19]. Figure 1A,B presents the sample analyses of CD200 and CD200R expression on B and T lymphocytes in patients with iPAH.

### 2.4. DNA Isolation and Calculation of EBV Load

The loads of EBV were evaluated as previously described [17]. According to the manufacturer’s instructions, the QIAamp DNA Blood Mini Kit (QIAGEN, Hilden, Germany) was utilized to isolate the DNA from 5 million PBMCs. The BioSpec-nano spectrophotometer (Shimadzu, Kyoto, Japan) was used to verify the concentration and purity of the isolated DNA. The number of EBV-DNA copies in the PBMCs was calculated with the use of the ISEX variant of the EBV polymerase chain reaction (PCR) kit (GeneProof, Brno, Czech Republic). The amplification of the certain conservative DNA sequence for the EBV nuclear antigen 1 (EBNA-1) gene resulted through real-time PCR. Nevertheless, the number of viral DNA copies per μL of eluent was calibrated for the efficiency of DNA isolation and to be expressed as the viral DNA copy number per μg of DNA. All of the samples were examined twice. The corresponding negative control (DNA elution buffer) was also incorporated. All samples below the detection threshold of 10 EBV DNA copies per μL were regarded as EBV negative [EBV(–)]. The PCR was settled with a 7300 Real-Time PCR System (Applied Biosystems, Foster City, CA, USA) [18].

### 2.5. Patients’ Infection Status Assessment

The presence of common viral, bacterial, and fungal pathogens was evaluated in all samples. The bacterial cultures (aerobic and non-aerobic) and fungal cultures were all carried out under standard housing conditions. No pathogens were found. The presence of the genetic material of hepatitis B virus (HBV), hepatitis C virus (HCV), *Herpes simplex* virus 1 and 2 (HSV-1 and -2), human immunodeficiency virus (HIV), human papillomavirus (HPV), cytomegalovirus (CMV), influenza virus, parvovirus B19, *Borrelia burgdorferi*, *Chlamydia pneumoniae*, *Chlamydia trachomatis*, *Toxoplasma gondii*, *Mycobacterium tuberculosis*, *Listeria* spp. and *Ureaplasma* spp., and was determined through applying the appropriate PCR-based test. None of the samples were positive [17,18,20].

### 2.6. Statistical Analysis

The statistical significance was determined with the nonparametric Mann-Whitney test. The *p* values below 0.05 were considered significant. The correlations between loads of EBV and several other variables were calculated via Spearman’s rank test and considered significant when the *p* value was below 0.05. All calculations were performed using Statistica 10 software (StatSoft, Tulsa, OK, USA) [18].

## 3. Results

### 3.1. Basic Clinical Parameters and Sex Characterizing the Studied Patients with Various Types of PAH and Persons from the Control Group

No statistically significant differences were found between the basic subpopulations of peripheral blood lymphocytes and the sex of patients with individual types of PAH. The sex of patients with CHD-PAH, CTD-PAH, CTEPH, and iPAH are presented in Table 1.

Basic clinical and laboratory parameters characterizing patients with selected types of PAH and persons from the control group are presented in Table 2.

No statistically significant relationships were found between the basic subpopulations of peripheral blood lymphocytes and selected clinical and laboratory parameters in patients with individual types of PAH.

A comparison of the percentage of regulatory T lymphocytes in selected types of PAH and the control group showed a significantly higher percentage of these lymphocytes in the group of patients with iPAH than in the control group (*p* <0.001). A significantly lower percentage of Treg was found in patients with CTEPH (*p* <0.001) and CHD-PAH (*p* <0.01) compared to iPAH.

### 3.2. Basic Hemodynamic Parameters Assessed during Cardiac Catheterization and Echocardiography in Patients with CHD-PAH, CTD-PAH, CTEPH, and iPAH

Basic hemodynamic parameters assessed during cardiac catheterization and echocardiography in patients with CHD-PAH, CTD-PAH, CTEPH, and iPAH are presented in Table 3.

### 3.3. Assessment of the Frequencies of Lymphocytes Expressing CD200 and CD200R Immunoregulatory Molecules in Patients with CHD-PAH, CTD-PAH, CTEPH, and iPAH and in the Control Group

Frequencies of lymphocytes expressing CD200 and CD200R immunoregulatory molecules in patients with CHD-PAH, CTD-PAH, CTEPH, and iPAH in the control group are presented in Table 4.

In accordance with the listed data, it can be seen that the percentage of T cells CD4+CD200+ was significantly higher in the iPAH group than in the control group (*p* < 0.001) and in the CTD-PAH group (*p* < 0.001). Patients with CHD-PAH (*p* < 0.001) and patients with CTEPH (*p* <0.01) also had a significantly higher percentage of T cells CD4+CD200+ compared to the control group.

A comparison of the percentage of T cells CD8+CD200+ in selected types of PAH and the control group showed the existence of a significantly higher percentage of these lymphocytes in the group of patients with iPAH than in the control group (*p* < 0.001) and in the group of patients with CTD-PAH (*p* < 0.01). CHD-PAH patients also had a higher percentage of T cells CD4+CD200+ than did the control group (*p* < 0.01).

Moreover, iPAH patients had a significantly higher percentage of B cells CD19+CD200+ than in the control group (*p* < 0.01). In addition, patients with iPAH, CHD-PAH, and CTEPH had a significantly lower percentage of T cells CD4+CD200R+ than in the control group (*p* < 0.001).

Compared to the control group, the patients of all types of PAH were characterized by a significantly lower percentage of T cells CD8+CD200R+: CHD-PAH and CTEPH (*p* < 0.001) and iPAH and CTD-PAH (*p* < 0.01).

The comparison of the percentage of B lymphocytes CD19+CD200R+ in the groups of patients with CHD-PAH, CTD-PAH, CTEPH, iPAH, and in the control group also revealed a significantly lower percentage of these lymphocytes in all studied PAH groups compared to the control group (*p* < 0.001).

No statistically significant relationships were found, however, between the expression of CD200 and CD200R molecules on the surface of T lymphocytes (CD4+ and CD8+) and B lymphocytes (CD19 +) and the analyzed clinical and laboratory parameters in patients with particular types of PAH.

### 3.4. Assessment of The Presence of EBV DNA in Individual Types of PAH and in the Control Group

In the CHD-PAH group, the presence of EBV DNA was demonstrated in 50% of all patients (13/26), while in the iPAH group, the presence of EBV DNA was indicated in 44% of all patients (11/25). In the CTEPH group, the presence of EBV DNA was evident in 60% of all patients (6/10) and in 33.3% of the CTD-PAH group (3/9). No EBV DNA was found in the control group.

In subsequent statistical processing, patients from the CHD-PAH and iPAH groups were divided, depending on whether EBV DNA was present, into EBV (+) and EBV (-) groups and a comparison was made between these groups and the control group (Table 5 and Table 6). In the CTEPH and CTD-PAH groups, no such division was made due to the small size of the groups.

## 4. Discussion

Research into the role of the immune response in the development and progression of PAH is important because the vascular remodeling of PAH eventually leads to impaired function through fibrosis and increased stiffness [21]. In the heterogeneity of PAH, both inflammatory and autoimmune components play a part [22].

Endothelial vascular cell dysfunction (which leads to PAH) is brought about by the imbalance between vasodilators and vasoconstrictors, activators and inhibitors of smooth muscle proliferation, pro- and anticoagulant factors, as well as pro- and anti-inflammatory factors [23].

The CD200/CD200R signaling pathway plays a critical role in regulating inflammatory response [24]. Accordingly, CD200 expression in cells causes a shift towards an activated phenotype. For example, CD200/CD200R cross-talk affects the Th1/Th2 balance resulting in increased cytokine production in Th2 cells [5]. Moreover, IL-2 and phytohemagglutinin (PHA) lymphocyte stimulation increase the expression of CD200 on CD4+ T-cells significantly more than on CD8+ T-cells [25]. The involvement of the CD200/CD200R signaling pathway in attenuating excessive immune response was demonstrated in a number of inflammatory conditions [26]. For instance, the association between the CD200R expression and inflammatory conditions was reported by Gao et al. who noted a negative correlation between CD200R expression on macrophages and C-reactive protein (CRP) levels in patients with rheumatoid arthritis [27]. Similarly, patients with sarcoidosis and decreased CD200R expression on monocytes had upregulated production of inflammatory cytokines [28]. However, the same inflammatory cytokines present at the inflammation site trigger CD200 expression, which attenuates the inflammatory response [16,25].

A correlation exists between the efficacy of CD200R-mediated inhibition of effector cell function and the receptor density on its surface. We noted that the low CD200R-expressing cells were barely inhibited by CD200R agonists and no inhibition was seen with very low expressing cells. This was confirmed by the finding of a reduced IL-8 secretion. On the other hand, in the literature, the immune response was more readily inhibited in the medium and high CD200R-expressing cells [11]. Here, the CD200-deficient mice had increased activated monocyte count. Similarly, the CD200R-deficiency was associated with upregulated production of tumor necrosis factor-alpha (TNF alpha) in response to lipopolysaccharide (LPS) stimulation and the inability to inhibit inflammatory cytokine production [29,30].

We saw that the plasma levels of soluble CD200 affect the expansion of T regulatory (Treg) cells, however, their role has not been fully explained to date [31]. Past research has shown that the anti-CD200 antibody-mediated blockade of the CD200-CD200R pathway results in a reduced Treg percentage [32]. What is more, a correlation between serum level of soluble CD200 (sCD200) and severity of dermatitis has been demonstrated. Having determined a correlation between soluble CD200 (sCD200) and IL-6 levels, sCD200 was proposed as an inflammatory marker. The sCD200 can block the CD200/CD200R interaction thus attenuating its immunosuppressive function [31]. Similarly, antibodies capable of blocking the CD200/CD200R cross-talk increased the severity of inflammation and tissue damage, whereas treatment with CD200R agonists reduced the severity of inflammation limiting tissue damage [33,34,35].

Walker et al. found that decreased CD200 and CD200R expression contributed to insufficient control of inflammation [30]. In their experimental inflammation model, they demonstrated that treatment of microglia with IL-4 increased the expression of CD200 and CD200R [24,36,37]. In other work, the IL-4 deficiency was shown to impair the expression of CD200 and CD200R in activated lymphocytes in patients with Alzheimer’s disease. A similar association between CD200R mRNA expression and IL-4 levels was demonstrated in children with different forms of epilepsy [38].

Our study showed that patients from PAH-CHD, CTEPH, and iPAH subsets had a significantly higher percentage of CD200-positive CD4+ and CD8+ T-cells than controls. In addition, patients from PAH-CHD and iPAH subsets had a significantly higher percentage of CD200-positive CD19+ T-cells than controls. However, the CD200R expression on lymphocytes was significantly lower across all PAH group subsets as compared to controls. In the light of the published data, our study appears to confirm the hypothesis that long-term exposure to cytokines results in high cellular expression of CD200. In line with previously reported findings, our study suggests that increased expression of CD200 is a compensatory mechanism that reduces excessive cytokine production [37]. Moreover, whilst the immunosuppressive effect of the CD200/CD200R signaling pathway seems to be somewhat inhibited in patients with PAH, the effective CD200/CD200R signaling pathway may actually counterbalance the effect of cytokine secretion. Additionally, the co-finding of CD200R-deficiency may indicate an association between PAH and the attenuated immunosuppressive effect of the CD200/CD200R signaling pathway. The main limitation of this study is the small number of patients with PAH, although PAH is known as a rare disease. Another limitation of this study is the lack of patients’ clinical follow-up, including hemodynamic assessment, echocardiographic exam, 6MWT, and CD200, and CD200R evaluation. The study group was heterogeneous, however, some studies indicate similar pathogenesis of changes in microcirculation in patients with PAH and CTEPH [39,40,41]. This study does not define a cause-effect relationship, although demonstrated results can be used in further studies.

## 5. Conclusions

CD200 and CD200R have important roles as negative co-stimulators in PAH-associated inflammation induction and persistence - notably that of iPAH. Moreover, dysregulation of the CD200/CD200R axis may be involved in the pathogeneses of several immune diseases. We believe CD200 and CD200R expression may serve to distinguish between PAH cases, hence, CD200 and CD200R usefulness as markers in PAH management needs further investigation.

## Figures and Tables

**Table 1:** The sex of patients with selected types of pulmonary arterial hypertension (PAH) and persons from the control group.

Group	Sex	Number of Patients/Controls	*p* Values for the Frequencies of CD4+CD200+ T Cells	*p* Values for the Frequencies of CD8+CD200+ T Cells	*p* Values for the Frequencies of CD19+CD200+ B Cells	*p* Values for the Frequencies of CD4+CD200R+ T Cells	*p* Values for the Frequencies of CD8+CD200R+ T Cells	*p* Values for the Frequencies of CD19+CD200R+ B Cells
CHD-PAH	Females	19	0.47	0.97	0.21	0.94	0.66	0.29
	Males	7						
CTD-PAH	Females	9	N/A	N/A	N/A	N/A	N/A	N/A
	Males	0						
CTEPH	Females	7	0.65	0.89	0.34	0.72	0.92	0.87
	Males	3						
iPAH	Females	15	0.31	0.80	0.46	0.93	0.49	0.76
	Males	10						
Control group	Females	12	0.61	0.27	0.35	0.68	0.60	0.11
	Males	8						

CHD-PAH: pulmonary arterial hypertension associated with congenital heart disease; CTD-PAH: pulmonary arterial hypertension associated with connective tissue disease; CTEPH: chronic thromboembolic pulmonary hypertension; iPAH: idiopathic pulmonary arterial hypertension; N/A: not applicable.

**Table 2:** Basic clinical and laboratory parameters characterizing patients with selected types of PAH and persons from the control group.

Parameter	Group	Median	Minimum	Maximum	Mean	SD	*p*
Age	CHD-PAH	57.5	23	81	55.69	17.34	CTEPH vs. CHD-PAH (*p* < 0.05), CTEPH vs. Control group (*p* < 0.05), CTEPH vs. CTD-PAH (*p* < 0.05), iPAH vs. CTEPH (*p* < 0.05),
	CTD-PAH	54	28	77	52.22	18.69	
	CTEPH	72.5	54	81	71.1	8.85	
	iPAH	62	23	81	56.52	17.23	
	Control group	56	39	77	58.05	11.12	
BMI	CHD-PAH	24.91	19.5	38.15	25.54	4.18	-
	CTD-PAH	22	20.32	27.98	23.79	2.96	
	CTEPH	23.67	20.44	35.04	24.74	4.18	
	iPAH	26	17.1	40.52	27.53	5.78	
	Control group	-	-	-	-	-	
6MWT [m]	CHD-PAH	378	50	578	323.15	149.64	-
	CTD-PAH	420	80	577.5	382.17	149.99	
	CTEPH	358.5	190	561	356.1	110.94	
	iPAH	374	136	556	377.84	99.38	
	Control group	-	-	-	-	-	
Neutrophils count [10^3^/mm^3^]	CHD-PAH	4.55	2.3	9.69	4.64	1.82	-
	CTD-PAH	4.29	2.14	7.91	4.8	1.7	
	CTEPH	4.64	1.74	9.01	5.01	2.24	
	iPAH	5.11	2.08	8.43	5.16	1.64	
	Control group	3.94	2.71	6.03	4.32	1.03	
Lymphocytes count [10^3^/mm^3^]	CHD-PAH	1.68	1.1	2.77	1.72	0.46	Control group vs. CHD-PAH (*p* ≤ 0.001), CTD-PAH vs. CHD-PAH (*p* ≤ 0.001), iPAH vs. CHD-PAH (*p* < 0.05), iPAH vs. CTD-PAH (*p* < 0.05)
	CTD-PAH	2.6	1.67	3.04	2.47	0.4	
	CTEPH	2.42	1.3	3.83	2.51	0.96	
	iPAH	2.01	1.2	3.14	2.15	0.56	
	Control group	2.54	1.53	3.07	2.44	0.45	
Hemoglobin concentration [g/dL]	CHD-PAH	15.1	7.4	22.1	15.4	4.38	-
	CTD-PAH	13.5	11.3	19.4	13.76	2.42	
	CTEPH	13.75	8.5	16.7	13.24	2.65	
	iPAH	13.6	9.5	18.5	13.69	2.04	
	Control group	14.35	12.5	15.6	14.31	0.86	
Platelets count [mm^3^]	CHD-PAH	156500	62000	299000	164115	64380	Control group vs. CHD-PAH (*p* ≤ 0.001), CTD-PAH vs. CHD-PAH (*p* < 0.05), iPAH vs. CHD-PAH (*p* < 0.05), CTD-PAH vs. Control group (*p* ≤ 0.001), CTEPH vs. Control group (*p* < 0.01), iPAH vs. Control group(*p* < 0.01), CTEPH vs. CTD-PAH (*p* < 0.05), iPAH vs. CTD-PAH (*p* ≤ 0.001)
	CTD-PAH	114000	55000	309000	147889	92369	
	CTEPH	182500	93000	348000	189000	73138	
	iPAH	213000	78000	474000	213800	80647	
	Control group	262500	186000	344000	263950	52744	
AspAT [U/L]	CHD-PAH	20	12	68	25.46	13.95	-
	CTD-PAH	32	17	38	27.67	7.79	
	CTEPH	27	19	127	36.4	32.22	
	iPAH	22	10	49	23.12	9.18	
	Control group	22.5	13	34	22.6	6.1	
ALAT [U/L]	CHD-PAH	16	26	22	22	16.87	Control group vs. CHD-PAH (*p* < 0.05), CTEPH vs. CHD-PAH (*p* < 0.01), CTEPH vs. CTD-PAH (*p* < 0.05), iPAH vs. CTEPH (*p* < 0.05)
	CTD-PAH	18	9	25.78	25.78	21.72	
	CTEPH	22.5	10	29.8	29.8	22.05	
	iPAH	18	25	20.04	20.04	10.41	
	Control group	18.5	20	19.75	19.75	7.49	
T CD3+ lymphocytes [%]	CHD-PAH	71.3	60.29	80.9	70.68	6.31	-
	CTD-PAH	70.53	57.31	80.45	70.29	6.85	
	CTEPH	69.8	63.1	81.62	71.07	6.3	
	iPAH	70.82	59.21	89.16	71.11	6.84	
	Control group	68.08	60.63	74.49	68.26	3.84	
B CD19+ lymphocytes [%]	CHD-PAH	9.36	2.46	26.25	9.76	4.89	
	CTD-PAH	9.05	2.79	21.99	10.18	6.29	
	CTEPH	11.25	5.36	16.91	11.01	4.14	
	iPAH	11.9	3.5	20.67	11.36	4.97	
	Control group	11.39	6.04	16.9	11.25	2.5	
NK cells (CD3-/CD16+CD56+) [%]	CHD-PAH	22.37	6.63	37.5	20.88	7.97	Control group vs. CHD-PAH (*p* < 0.05). CTD-PAH vs. CHD-PAH (*p* ≤ 0.001). CTEPH vs. CHD-PAH (*p* ≤ 0.001). iPAH vs. CHD-PAH (*p* ≤ 0.001). CTD-PAH vs. Control group (*p* ≤ 0.001). CTEPH vs. Control group (*p* < 0.05). iPAH vs. Control group (*p* ≤ 0.001)
	CTD-PAH	10.9	2.34	19.77	10.82	5.76	
	CTEPH	8.93	4.32	17.41	9.65	4.6	
	iPAH	11.23	3.99	20.43	11.1	4.22	
	Control group	14.43	12.16	19.34	15.35	2.25	
NKT-like cells CD3+CD16+CD56+ [%]	CHD-PAH	1.58	0.24	8.47	2.62	2.31	CHD-PAH vs. iPAH (*p* < 0.01)
	CTD-PAH	1.04	0.21	8.2	2.97	3.08	
	CTEPH	3.17	0.77	11.26	4.1	3.68	
	iPAH	5.23	0.67	10.94	5.26	2.67	
	Control group	3.27	1.15	4.92	3.02	1.02	
T CD4+/CD3+ lymphocytes [%]	CHD-PAH	42.16	21.58	57.43	41.25	10	CTD-PAH vs. Control group (*p* < 0.01). CTEPH vs. CTD-PAH (*p* < 0.01)
	CTD-PAH	35.54	28.43	59.88	39.84	10.22	
	CTEPH	45.37	39.14	51.33	45.42	4.47	
	iPAH	36.91	19.73	62.92	38.68	13.42	
	Control group	44.16	40.71	48.84	44.46	2.5	
T CD8+/CD3+ lymphocytes [%]	CHD-PAH	27.21	12.78	47.16	26.94	8.2	Control group vs. CHD-PAH (*p* ≤ 0.001). CTEPH vs. Control group (*p* < 0.01). iPAH vs. Control group (*p* ≤ 0.001)
	CTD-PAH	30.62	10.18	39.87	28	10.82	
	CTEPH	20.29	11.17	36.94	23.02	7.67	
	iPAH	28.3	9.19	59.29	29.46	14.17	
	Control group	34.73	29.33	39.6	34.36	3.29	
T CD4+: T CD8+ lymphocytes ratio	CHD-PAH	1.62	0.46	4.49	1.78	0.95	Control group vs. CTEPH (*p* < 0.05), CTD-PAH vs. CTEPH (*p* < 0.05)
	CTD-PAH	1.31	0.87	4.86	1.84	1.38	
	CTEPH	2.09	1.09	4.5	2.24	0.97	
	iPAH	1.25	0.34	6.85	1.95	1.78	
	Control group	1.29	1.03	1.57	1.31	0.16	
T regulatory cells [%]	CHD-PAH	7.43	4.67	15.59	8.43	2.87	CHD-PAH vs. CTEPH (*p* < 0.01), Control group vs. CTEPH (*p* < 0.05), CTD-PAH vs. CTEPH (*p* < 0.05), CHD-PAH vs. iPAH (*p* < 0.01), Control group vs. iPAH (*p* ≤ 0.001), CTD-PAH vs. iPAH (*p* < 0.05), CTEPH vs. iPAH (*p* ≤ 0.001)
	CTD-PAH	7.25	4.73	11.59	8.11	2.39	
	CTEPH	4.13	1.79	10.33	4.65	2.55	
	iPAH	11.21	5.94	23.81	11.98	3.97	
	Control group	7.37	3.15	10.15	7.1	1.94	
NT-proBNP [pg/mL]	CHD-PAH	836	106.8	9350	1597.66	1930.48	-
	CTD-PAH	1279	429	4015	1530	1384.07	
	CTEPH	1756.5	53	5991	2071.52	1617.33	
	iPAH	1546	210	10144	1940.22	2072.29	
	Control group	-	-	-	-	-	

6MWT: 6-min walk test; ALAT: alanine transaminase; AspAT: aspartate transaminase; BMI: body mass index; CHD-PAH: pulmonary arterial hypertension associated with congenital heart disease; CTD-PAH: pulmonary arterial hypertension associated with connective tissue disease; CTEPH: chronic thromboembolic pulmonary hypertension; iPAH: idiopathic pulmonary arterial hypertension; NK: natural killer; SD: standard deviation.

**Table 3:** Basic hemodynamic parameters assessed during cardiac catheterization and echocardiography in patients with CHD-PAH, CTD-PAH, CTEPH, and iPAH.

Parameter	Group	Median	Minimum	Maximum	Mean	SD	*p*
Pulmonary vascular resistance (PVR) [dyne / s / cm^-5^]	CHD-PAH	838.62	134	2803	1066.32	696.02	CHD-PAH vs. CTD-PAH (*p* ≤ 0.001). CTD-PAH vs. CTEPH (*p* < 0.05). CTD-PAH vs. iPAH (*p* < 0.05)
	CTD-PAH	355	139	1292	424.53	369.55	
	CTEPH	715.5	305.51	1125.8	720.4	234.12	
	iPAH	651	158	1599	697.6	314.62	
	Control group	-	-	-	-	-	
Cardiac index (CI) [L / min / m^2^]	CHD-PAH	2.27	1.65	7.32	2.52	1.08	CTEPH vs. CHD-PAH (*p* < 0.05). CTEPH vs. CTD-PAH (*p* < 0.05). iPAH vs. CTEPH (*p* < 0.01)
	CTD-PAH	3.21	1.83	4.67	3.16	0.83	
	CTEPH	2.05	1.75	5.8	2.53	1.25	
	iPAH	2.6	1.43	3.75	2.54	0.65	
	Control group	-	-	-	-	-	
Cardiac output (CO) [L / min]	CHD-PAH	3.84	2.29	13.9	4.2	2.11	CTD-PAH vs. CHD-PAH (*p* ≤ 0.001). iPAH vs. CHD-PAH (*p* < 0.01). CTEPH vs. CTD-PAH (*p* < 0.01). iPAH vs. CTEPH (*p* < 0.05)
	CTD-PAH	5.75	3.02	8.47	5.37	1.57	
	CTEPH	3.56	2.48	9.51	4.19	2.17	
	iPAH	4.46	2.11	6.42	4.64	1.15	
	Control group	-	-	-	-	-	
Mean right atrial pressure (mRAP) [mmHg]	CHD-PAH	8	1	16	7.88	3.25	-
	CTD-PAH	8	3	15	8.56	4.03	
	CTEPH	9	3	18	9.5	5.02	
	iPAH	9	2	23	8.8	5.69	
	Control group	-	-	-	-	-	
Mean pulmonary artery pressure (mPAP) [mmHg]	CHD-PAH	48.5	26	106	53.61	22.76	CHD-PAH vs. CTD-PAH (*p* < 0.01). CTD-PAH vs. CTEPH (*p* < 0.05). CTD-PAH vs. iPAH (*p* < 0.05)
	CTD-PAH	34	25	68	35.3	13.25	
	CTEPH	46	25.6	56	45.66	8.75	
	iPAH	48	25	66	45.56	12.03	
	Control group	-	-	-	-	-	
Mean pulmonary artery pressure by echocardiography (PASP) [mmHg]	CHD-PAH	76.5	41	150	82.15	29.02	CTD-PAH vs. CHD-PAH (*p* < 0.01). CTEPH vs. CTD-PAH (*p* < 0.05). iPAH vs. CTD-PAH (*p* < 0.05)
	CTD-PAH	57	35	110	58.22	21.68	
	CTEPH	78.5	39	110	80.4	21.57	
	iPAH	77	37	105	72.2	18.92	
	Control group	-	-	-	-	-	
Mean pressure in the right ventricle on echocardiography [mmHg]	CHD-PAH	72	40	150	81.19	29.69	CHD-PAH vs. CTD-PAH (*p* < 0.05). CTD-PAH vs. CTEPH (*p* < 0.05).
	CTD-PAH	56	36	115	58.67	24.07	
	CTEPH	76.5	50	110	79.7	18.54	
	iPAH	76	42	96	68.84	17.66	
	Control group	-	-	-	-	-	

CHD-PAH: pulmonary arterial hypertension associated with congenital heart disease; CTD-PAH: pulmonary arterial hypertension associated with connective tissue disease; CTEPH: chronic thromboembolic pulmonary hypertension; iPAH: idiopathic pulmonary arterial hypertension; SD: standard deviation.

**Table 4:** Basic laboratory markers characterizing patients with selected types of PAH and persons from the control group.

Parameter	Group	Median	Minimum	Maximum	Mean	SD	*p*
T lymphocytes CD4+CD200+ [%]	CHD-PAH	10.48	3.47	15.1	10.79	3.11	CHD-PAH vs. Control group (*p* ≤ 0.001). CHD-PAH vs. CTD-PAH (*p* < 0.05). Control group vs. CTEPH (*p* < 0.01). Control group vs. iPAH (*p* ≤ 0.001). CTD-PAH vs. iPAH (*p* ≤ 0.001)
	CTD-PAH	6.84	4.28	7.45	6.27	1.12	
	CTEPH	10.13	7.7	12.96	10.39	2.29	
	iPAH	16.45	5.6	34.65	16.78	7.15	
	Control group	4.79	1.05	9.03	5.12	2.43	
T lymphocytes CD4+CD200R+ [%]	CHD-PAH	13.14	3.23	40.71	15.33	9.28	Control group vs. CHD-PAH (*p* ≤ 0.001). CTD-PAH vs. Control group (*p* < 0.05). CTEPH vs. Control group (*p* ≤ 0.001). iPAH vs. Control group (*p* ≤ 0.001)
	CTD-PAH	15.8	7.41	27.97	16.55	6.93	
	CTEPH	12.86	3.96	21.28	12.59	6.35	
	iPAH	11.93	2.04	18.36	11.43	5.07	
	Control group	22.27	7.39	38.83	23.11	7.95	
T lymphocytes CD8+CD200+ [%]	CHD-PAH	6.45	2.33	16.58	8.52	4.94	CHD-PAH vs. Control group (*p* < 0.01). Control group vs. CTEPH (*p* < 0.05). Control group vs. iPAH (*p* ≤ 0.001). CTD-PAH vs. iPAH (*p* < 0.01)
	CTD-PAH	4.59	1.88	8.29	5.34	2.23	
	CTEPH	7.54	2.73	18.92	8.51	5.26	
	iPAH	10.45	3.47	28.04	12.66	6.62	
	Control group	3.64	0.35	6.45	3.88	1.56	
T lymphocytes CD8+CD200R+ [%]	CHD-PAH	8.57	1.63	36.84	9.29	6.88	Control group vs. CHD-PAH (*p* ≤ 0.001). CTD-PAH vs. Control group (*p* < 0.01). CTEPH vs. Control group (*p* ≤ 0.001). iPAH vs. Control group (*p* < 0.01)
	CTD-PAH	8.41	3.6	13.76	8.25	3.55	
	CTEPH	6.36	0.59	16.17	7.18	5.65	
	iPAH	8.77	1.9	18.91	9.51	4.26	
	Control group	13.23	6.03	31.92	14.38	5.78	
B lymphocytes CD19+CD200+ [%]	CHD-PAH	83.41	60.39	99.95	82.71	11.69	Control group vs. CHD-PAH (*p* < 0.05). iPAH vs. Control group (*p* < 0.01). iPAH vs. CTD-PAH (*p* < 0.05)
	CTD-PAH	74.38	63.5	93.97	76.46	10.8	
	CTEPH	82.27	69.27	99.99	82.91	11.48	
	iPAH	85.92	0	99.99	82.36	18.84	
	Control group	76.59	67.39	93.19	76.26	6.58	
B lymphocytes CD19+CD200R+ [%]	CHD-PAH	10.66	5.25	19.19	10.73	3.3	Control group vs. CHD-PAH (*p* ≤ 0.001). CTD-PAH vs. Control group (*p* ≤ 0.001). CTEPH vs. Control group (*p* ≤ 0.001). iPAH vs. Control group (*p* ≤ 0.001)
	CTD-PAH	8.14	4.15	18.04	9.96	4.9	
	CTEPH	11.14	6.02	17.83	11.31	3.96	
	iPAH	11.19	3.92	20.05	11.09	3.52	
	Control group	24.8	16.91	31.89	25.02	4.06	

CHD-PAH: pulmonary arterial hypertension associated with congenital heart disease; CTD-PAH: pulmonary arterial hypertension associated with connective tissue disease; CTEPH: chronic thromboembolic pulmonary hypertension; iPAH: idiopathic pulmonary arterial hypertension; SD: standard deviation.

**Table 5:** Percentage of lymphocytes expressing CD200 and CD200R molecules in patients with CHD-PAH divided into EBV (+) and EBV (-) groups.

Parameter	Group	Median	Minimum	Maximum	Mean	SD	*p*
T CD4+CD200+ lymphocytes [%]	CHD-PAH EBV+	9.67	8.69	14.18	10.5	2.01	CHD-PAH EBV- vs. Control group (*p* ≤ 0.001). CHD-PAH EBV+ vs. Control group (*p* ≤ 0.001).
	CHD-PAH EBV-	12.31	3.47	15.1	11.03	3.88	
	Control group	4.79	1.05	9.03	5.12	2.43	
T CD4+CD200R+ lymphocytes [%]	CHD-PAH EBV+	13.1	3.23	36.15	15.96	8.98	Control group vs. CHD-PAH EBV- (*p* < 0.01). Control group vs. CHD-PAH EBV+ (*p* < 0.01)
	CHD-PAH EBV-	13.64	3.89	40.71	14.79	9.84	
	Control group	22.27	7.39	38.83	23.11	7.95	
T CD8+CD200+ lymphocytes [%]	CHD-PAH EBV+	5.42	2.85	16.58	8.54	5.82	CHD-PAH EBV- vs. Control group (*p* < 0.01). CHD-PAH EBV+ vs. Control group (*p* < 0.01).
	CHD-PAH EBV-	6.83	2.33	15.02	8.51	4.27	
	Control group	3.64	0.35	6.45	3.88	1.56	
T lymphocytes CD8+CD200R+ [%]	CHD-PAH EBV+	10.66	1.63	13.89	9.11	4.32	CHD-PAH EBV- vs. Control group (*p* < 0.01). CHD-PAH EBV+ vs. Control group (*p* < 0.05)
	CHD-PAH EBV-	6.75	2.75	36.84	9.45	8.68	
	Control group	13.23	6.03	31.92	14.38	5.78	
B CD19+CD200+ lymphocytes [%]	CHD-PAH EBV+	85.3	64.62	99.95	86.7	10.46	Control group vs. CHD-PAH EBV+ (*p* < 0.01)
	CHD-PAH EBV-	79.02	60.39	99.45	79.3	11.96	
	Control group	76.59	67.39	93.19	76.26	6.58	
B CD19+CD200R+ lymphocytes [%]	CHD-PAH EBV+	10.29	6.73	12.82	9.74	2.26	Control group vs. CHD-PAH EBV- (*p* ≤ 0.001). Control group vs. CHD-PAH EBV+ (*p* ≤ 0.001)
	CHD-PAH EBV-	11.1	5.25	19.19	11.58	3.87	
	Control group	24.8	16.91	31.89	25.02	4.06	

CHD-PAH: pulmonary arterial hypertension associated with congenital heart disease; EBV: Epstein-Barr virus; SD: standard deviation.

**Table 6:** Percentage of lymphocytes expressing CD200 and CD200R molecules in patients with iPAH, divided into EBV (+) and EBV (-) groups.

Parameter	Group	Median	Minimum	Maximum	Mean	SD	*p*
T CD4+CD200+ lymphocytes [%]	iPAH EBV+	15.48	6.03	24.2	14.96	5.2	iPAH EBV- vs. Control group (*p* ≤ 0.001). iPAH EBV+ vs. Control group (*p* ≤ 0.001)
	iPAH EBV-	18.38	5.6	34.65	18.21	8.29	
	Control group	4.79	1.05	9.03	5.12	2.43	
T CD4+CD200R+ lymphocytes [%]	iPAH EBV+	11.17	2.04	18.36	10	5	iPAH EBV- vs. Control group (*p* ≤ 0.001). iPAH EBV+ vs. Control group (*p* ≤ 0.001)
	iPAH EBV-	13.76	2.9	18.34	12.55	5	
	Control group	22.27	7.39	38.83	23.11	7.95	
T CD8+CD200+ lymphocytes [%]	iPAH EBV+	10	4.16	25.11	11	5.75	iPAH EBV- vs. Control group (*p* ≤ 0.001).
	iPAH EBV-	12.91	3.47	28.04	13.96	7.17	
	Control group	3.64	0.35	6.45	3.88	1.56	
T lymphocytes CD8+CD200R+ [%]	iPAH EBV+	9.99	5.52	16.43	10.29	3.32	iPAH EBV+ vs. Control group (*p* ≤ 0.01)
	iPAH EBV-	8.21	1.9	18.91	8.91	4.92	
	Control group	13.23	6.03	31.92	14.38	5.78	
B CD19+CD200+ lymphocytes [%]	iPAH EBV+	85.56	69.24	99.99	84.66	7.59	iPAH EBV- vs. Control group (*p* < 0.01)
	iPAH EBV-	86.94	0	99.18	80.54	24.56	
	Control group	76.59	67.39	93.19	76.26	6.58	
B CD19+CD200R+ lymphocytes [%]	iPAH EBV+	11.8	7.64	20.05	12.1	3.37	iPAH EBV- vs. Control group (*p* ≤ 0.001).
	iPAH EBV-	10.16	3.92	16.14	10.29	3.54	
	Control group	24.8	16.91	31.89	25.02	4.06	

EBV: Epstein-Barr virus; iPAH: idiopathic pulmonary arterial hypertension; SD: standard deviation.

## Data Availability

Due to privacy and ethical concerns, the data that support the findings of this study are available on request from the First Author, [M.T.].
